# Understanding the Nuanced Concept of Hemodynamic Incoherence and Its Underlying Physiology

**DOI:** 10.3390/jcm15155811

**Published:** 2026-07-24

**Authors:** Philippe Rola, Ashley Miller, Korbin Haycock, Rory Spiegel, Kiran Rikhraj, Eduardo Kattan, Glenn Hernández

**Affiliations:** 1 Intensive Care Unit, Santa Cabrini Hospital ICU CEMTL, University of Montreal, Montreal, QC H1T 1P7, Canada; 2 Department of Intensive Care Medicine and Anaesthesia, Shrewsbury and Telford Hospitals NHS Trust, Shrewsbury SY3 8XQ, UK; 3 Department of Emergency Medicine, Riverside University Health System, Moreno Valley, CA 92555, USA; 4 Department of Emergency Medicine, Department of Critical Care, Medstar Hospital Center, Washington, DC 20010, USA; 5 Division of Critical Care Medicine, Vancouver General Hospital, University of British Columbia, Vancouver, BC V5Z 1N1, Canada; 6 Departamento de Medicina Intensiva, Facultad de Medicina, Pontificia Universidad Católica de Chile, Santiago 8331150, Chile; e.kattan@gmail.com (E.K.);

**Keywords:** hemodynamics, shock, critical care, tissue perfusion, sepsis

## Abstract

Hemodynamic incoherence is commonly defined as a dissociation between normalization of macrocirculatory variables and persistent impairment of tissue perfusion. However, this phenomenon is not physiologically homogeneous. We propose a conceptual distinction between two forms of macro–microcirculatory incoherence. Type 1 incoherence represents primary microcirculatory dysfunction, in which intrinsic alterations in microvascular regulation and flow distribution lead to heterogeneous capillary perfusion despite apparently adequate upstream hemodynamics, as observed in sepsis. In contrast, Type 2 incoherence reflects secondary impairment of microcirculatory flow resulting from hemodynamic constraints elsewhere in the circulation, including excessive vasoconstriction, limited cardiac forward flow, or venous congestion. In this setting, impaired perfusion arises from ineffective transmission of flow across the system rather than primary microvascular failure. The four-interface model of the circulation provides a unifying physiological framework to interpret these mechanisms as failures of energy transfer across sequential hemodynamic domains. Recognizing these distinct forms of incoherence may improve not only physiologic interpretation of shock and help avoid conflating fundamentally different mechanisms under a single conceptual entity, but also be key in individualizing therapeutic interventions to the proper phenotypes of tissue malperfusion.

## 1. Introduction

Shock has historically been defined as a state of oxygen delivery inadequate to meet metabolic demand, resulting in tissue hypoxia and organ dysfunction. Clinical management has traditionally focused on correcting macrocirculatory variables such as mean arterial pressure, cardiac output, and systemic vascular resistance. However, multiple clinical and experimental observations have demonstrated that normalization of these macrocirculatory parameters does not consistently translate to restoration of tissue perfusion [1,2].

In a recently proposed conceptual framework, the circulation can be viewed as a series of functional transitions, or interfaces, through which energy and blood flow must be effectively transmitted in order to achieve adequate tissue perfusion [3]. In this context, macro–microcirculatory coupling refers to the effective transmission of flow across these interfaces, rather than a simple association between pressure variables.

These four hemodynamic interfaces are defined as:

Interface I: Left ventricle–systemic arterial circulation;

Interface II: Arteriolar–capillary circulation;

Interface III: Capillary–venular drainage;

Interface IV: Right ventricle–pulmonary circulation.

Each interface represents a potential site where circulatory energy transfer may become impaired or uncoupled. Importantly, dysfunction at different interfaces may produce similar clinical signs of hypoperfusion despite fundamentally different physiologic mechanisms. In addition, capillary circulation is characterized by transcapillary exchange with the interstitial compartment and a parallel return of capillary flux via the lymphatic and venous systems, both of which are integral to effective tissue perfusion.

The purpose of the present article is to propose a classification of macro–microcirculatory uncoupling based on physiological mechanisms.

The originality of the proposed classification lies not in redescribing the existence of macro–microcirculatory dissociation, but in separating physiologically distinct mechanisms currently grouped under that term. Previous descriptions of hemodynamic incoherence have predominantly identified persistent microcirculatory impairment despite normalization of systemic variables. We distinguish primary microvascular dysfunction, termed Type 1 incoherence, from secondary microcirculatory impairment caused by constraints elsewhere in the circulation, termed Type 2 incoherence. By mapping the latter to failures at one or more of four hemodynamic interfaces, the framework moves from recognizing dissociation to identifying its dominant mechanism and potentially modifiable cause.

## 2. Macro–Microcirculatory Coherence

Hemodynamic coherence describes the effective coupling between systemic hemodynamics and tissue perfusion. In a coherent circulation, changes in cardiac output, arterial pressure, and vascular tone are translated into appropriate microvascular perfusion, while local autoregulatory mechanisms distribute flow according to regional metabolic demand. Coherence therefore does not imply a simple proportional relationship between macrocirculatory variables and capillary blood flow. Rather, it implies that systemic hemodynamics are sufficient to support regulated microvascular perfusion across a range of physiological conditions. This concept has also been described as macro–microcirculatory coupling, while loss of this relationship is described as macro–microcirculatory uncoupling or hemodynamic incoherence.

This distinction is clinically important because similar macrocirculatory values may arise from very different underlying physiological states [4]. A mean arterial pressure of 70 mmHg with preserved forward flow, low venous pressure, and improving capillary refill time is not equivalent in terms of tissue perfusion to the same arterial pressure achieved with high-dose vasopressor in a patient with falling stroke volume, rising venous pressure, and worsening mottling. The issue is therefore not simply whether macrocirculatory variables appear normal, but whether they are associated with appropriate tissue perfusion. In shock states this coherence may be lost. Restoration of systemic hemodynamic variables does not necessarily result in normalization of microvascular perfusion. Direct visualization of the microcirculation has demonstrated that microvascular perfusion abnormalities may persist even after normalization of arterial pressure and cardiac output [5].

Clinical investigations by Ospina-Tascón and colleagues have further demonstrated that early improvement in microcirculatory flow is associated with improved outcomes in septic shock, whereas persistent microcirculatory abnormalities despite macrocirculatory stabilization are associated with worse prognosis [6]. More broadly, association and dissociation between the macro- and microcirculation have been described across critically ill patients with shock [7].

Within the four-interface framework, loss of hemodynamic coherence can be understood as a failure of effective flow transmission between upstream macrocirculatory interfaces and the microvascular domain. Furthermore, derangements in the venous circulation can have important effects on the microcirculatory conditions promoting perfusion.

“Hemodynamic coherence” is defined as the effective translation of systemic hemodynamics into tissue perfusion. “Hemodynamic incoherence” is now used as the principal term for failure of this relationship. “Macro–microcirculatory uncoupling” is retained as a physiologically descriptive synonym, while “dissociation” is used mainly when referring to terminology from the existing literature. “Interface uncoupling” is reserved for dysfunction occurring at a specific hemodynamic interface.

**Figure 1:**
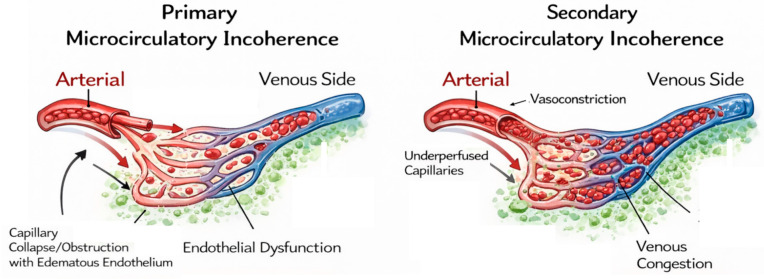
Schematic illustration of the two types of microcirculatory incoherence. On the left, endothelial pathology results in incoherence, while on the right, uncoupling at Interface I, II or III results in decreased perfusion.

There are two distinct ways in which this uncoupling can occur, and the distinction is important because one reflects primary microcirculatory dysfunction, whereas the other reflects secondary impairment due to upstream or downstream hemodynamic constraints (Figure 1).

### 2.1. Type 1: Primary Microcirculatory Incoherence

In Type 1 incoherence, macrocirculatory variables may appear adequate, yet perfusion at the level of the capillary bed remains impaired due to abnormalities in arteriolar regulation or microvascular flow distribution.

This pattern is most commonly observed in septic shock, where endothelial dysfunction, glycocalyx degradation, leukocyte adhesion, and impaired microvascular autoregulation lead to heterogeneous perfusion within the capillary network. Some capillaries receive excessive flow while others remain poorly perfused or stagnant. Indeed, acute hemodynamic tests, such as those mimicking a transient increase in flow or pressure, can disclose the heterogeneity of response of tissue perfusion. Matthias Jacquete Lagreze et al. showed that after a passive leg raising maneuver, an increase in CO was not always accompanied by a reduction in capillary refill time (CRT) [8]. In fact, it is not infrequent that patients displayed paradoxical responses, both in laser Doppler flowmetry or quantitative CRT [9]. On the other hand, Hernandez et al. found wide dispersion of CRT response during fluid challenges (flow) in preload responsive patients or mean arterial pressure challenges (pressure), with some patients showing deteriorating tissue perfusion in the latter [10]. These acute hemodynamic challenges were tested in the recent ANDROMEDA-SHOCK-2 trial, which showed an overall effectiveness of CRT normalization in approximately 50% of the patients [11].

Within the interface framework, this corresponds predominantly to dysfunction of the arteriolar–capillary–venular microvascular unit. Although centred on Interface II, it extends into the capillary and post-capillary venular compartments, producing impaired distribution of flow within the microvascular network despite apparently adequate upstream conditions.

### 2.2. Type 2: Secondary Microcirculatory Incoherence

Incoherence or macro–microcirculatory uncoupling may also occur when upstream or downstream hemodynamic constraints prevent effective transmission of flow across the interfaces, despite apparently adequate macrocirculatory variables.

This may arise through two broad mechanisms. First, initiation or escalation of vasopressor therapy may produce excessive vasoconstriction. By increasing tone in small resistance vessels, vasopressors may increase the local closing pressure of arteriolar segments, so that some vascular units become vulnerable to intermittent loss of patency despite an apparently adequate upstream arterial pressure [12]. This may produce heterogeneous microvascular flow dropout and uncoupling at Interface II. In parallel, increased systemic vascular tone may increase left ventricular afterload and impair forward flow, contributing to uncoupling at Interface I [12]. Second, increases in mean systemic filling pressure that do not translate into effective forward flow may result in venous congestion and uncoupling at Interface III and/or IV. When congestion is associated with impaired cardiac output, microvascular perfusion may be limited by both reduced forward flow and elevated downstream venous pressure. However, venous congestion may also occur despite preserved global cardiac output. In this setting, total systemic capillary flow is not necessarily reduced, because venous return and cardiac output remain equal at steady state. Instead, elevated venous pressure increases capillary hydrostatic pressure, promotes transcapillary filtration, and may overwhelm lymphatic clearance. The resulting interstitial edema increases diffusion distance and may externally constrain small vessels and lymphatics, particularly in low-compliance tissues or encapsulated organs, producing regional heterogeneity of microvascular perfusion and exchange.

In these settings, increases in arterial pressure do not necessarily translate into improved tissue perfusion, because microvascular perfusion may remain constrained by low forward flow, elevated venous pressure, tissue edema, impaired drainage, or heterogeneous regional flow distribution.

Thus, the apparent loss of coherence does not necessarily reflect intrinsic microcirculatory failure. Instead, it may reflect secondary impairment of tissue perfusion due to constraints at other hemodynamic interfaces. This distinction is clinically important because the therapeutic approach may differ substantially. Type 1 incoherence is primarily managed by treating the underlying inflammatory, infectious, endothelial, or rheological process, while avoiding harmful escalation of macrocirculatory targets. Type 2 incoherence may instead require correction of the dominant hemodynamic constraint, such as reducing excessive vasoconstriction, improving forward flow, unloading venous congestion, or optimizing ventricular–arterial or ventricular–pulmonary coupling.

## 3. Mechanistic Phenotypes Rather than Fixed Categories

These categories should be understood as mechanistic phenotypes rather than mutually exclusive diagnostic labels. Many patients, particularly those with septic shock, may move between phenotypes over time or express several simultaneously. Primary microvascular dysfunction, excessive vasoconstrictive tone, myocardial dysfunction, right ventricular–pulmonary vascular uncoupling, and venous congestion may coexist in the same patient. The purpose of this classification is therefore not to force patients into rigid groups, but to identify the dominant treatable constraint to tissue perfusion at a given moment.

## 4. Minimal Bedside Evaluation of the Four Interfaces

The framework is intended for patients with persistent or unexplained signs of impaired tissue perfusion during shock resuscitation, particularly when arterial pressure and other global hemodynamic variables appear acceptable or when the response to treatment is discordant. Although septic shock provides the most familiar setting for Type 1 incoherence, the classification is applicable to cardiogenic, obstructive, hypovolemic, vasoplegic, and mixed shock states. It may be used in emergency, intensive care, and perioperative environments wherever serial clinical examination and focused hemodynamic assessment are available. Its purpose is not to mandate a fixed monitoring protocol, but to structure repeated assessment of the dominant physiological constraint.

The four-interface model can be translated into a rapid physiologic bedside examination using simple clinical observations and focused ultrasound assessment.

Interface I (Left ventricle–arterial system): Visual ejection fraction estimation and assessment of forward flow such as left ventricular outflow tract velocity time interval (LVOT VTI).

Interface II (Arteriolar–capillary): Capillary refill time, mottling, and where available, assessment of microvascular reactivity (e.g., laser Doppler or vascular occlusion testing) or vascular microscopy.

Interface III (Capillary–venular): Assessment of venous congestion using the inferior vena cava, central venous pressure, jugular venous pressure, and where available, Doppler evaluation of venous flow patterns such as hepatic, intrarenal and/or portal veins, and the venous excess ultrasound score (VExUS) [13].

Interface IV (Right ventricle–pulmonary circulation): Right ventricular size and function (e.g., tricuspid annular plane systolic excursion or TAPSE) and septal position/dynamics on echocardiography.

These are not exhaustive as a review of all hemodynamic measures is beyond the scope of this article, and can also involve different technologies. In addition, global markers of organ perfusion such as mentation, urine output, and lactate provide important complementary information but reflect organ congestion consequences rather than interface-specific function (Table 1).

## 5. Illustrative Clinical Cases

### 5.1. Type 1 Hemodynamic Incoherence: Primary Microcirculatory Dysfunction

A 68-year-old man was admitted with septic shock secondary to pneumonia. On initial assessment, he was febrile, tachycardic, and hypotensive with diffuse warm extremities and prolonged capillary refill time (CRT) of approximately 5 s. Lactate was elevated at 4.8 mmol/L. Point-of-care ultrasound demonstrated a hyperdynamic left ventricle with small ventricular cavities and a collapsible inferior vena cava, suggesting preload responsiveness and preserved macrocirculatory reserve.

Initial resuscitation included fluid administration, broad-spectrum antibiotics, and norepinephrine titration. Over the following hour, mean arterial pressure increased from 55 mmHg to 72 mmHg, while cardiac output assessed by LVOT velocity time integral also increased substantially following a passive leg raise maneuver. Despite these apparent improvements in macrocirculatory variables, peripheral perfusion remained abnormal, with persistent prolongation of CRT and mottling. Repeat bedside assessment suggested marked heterogeneity of tissue perfusion, with some skin territories appearing hyperemic while others remained poorly perfused.

Interface analysis: The dominant abnormality is located at Interface II, the arteriolar–capillary transition. Interface I appears relatively preserved or even hyperdynamic, as suggested by preserved cardiac output reserve and an increase in LVOT VTI after passive leg raising. Interface IV is not the dominant limitation in this phenotype if right ventricular size and function are preserved. Interface III may contribute if venous congestion develops later, but given the non-plethoric IVC, the primary problem is failure of arteriolar–capillary flow distribution. Thus, the apparent macro–microcirculatory dissociation is not due to inadequate upstream cardiac energy generation, but to impaired transmission and distribution of flow within the microvascular network itself.

This pattern illustrates Type 1 hemodynamic incoherence, in which restoration of upstream macrocirculatory parameters fails to normalize microvascular perfusion because the primary dysfunction resides within the microcirculatory network itself. In septic shock, endothelial injury, glycocalyx degradation, leukocyte adhesion, altered rheology, and impaired autoregulatory vasomotor responses contribute to heterogeneous capillary recruitment and maldistribution of flow. Under these conditions, increases in cardiac output or arterial pressure may not translate into proportional improvement in tissue perfusion.

Within the four-interface framework, this phenomenon corresponds primarily to dysfunction at Interface II, the arteriolar–capillary transition. Importantly, the issue is not an absence of circulatory energy generation, but rather an inability to distribute flow effectively across the capillary bed. Some capillary units may receive excessive flow while adjacent territories remain intermittently perfused or stagnant.

Recognition of this phenotype is clinically important because further escalation of macrocirculatory targets alone may fail to restore coherence and may potentially worsen downstream physiology. In such cases, definitive management depends primarily on reversal of the underlying inflammatory and endothelial pathology through source control, antimicrobial and perhaps anti-inflammatory therapy, disease-specific molecules and time-dependent recovery of microvascular function, rather than indefinite escalation of pressure or flow targets alone.

Take-home message: Persistent hypoperfusion despite restored pressure and recruitable forward flow suggests primary failure of microvascular distribution rather than an unresolved macrocirculatory deficit.

### 5.2. Type 2 Hemodynamic Incoherence: Excessive Vasoconstrictive Constraint

A 74-year-old man with ischemic cardiomyopathy and pneumonia presented with hypotension and respiratory distress. On admission, blood pressure was 78/52 mmHg with a heart rate of 118 beats/min. Extremities were cool and capillary refill time (CRT) was approximately 4 s. Point-of-care ultrasound demonstrated moderately reduced left ventricular systolic function with a left ventricular outflow tract velocity time integral (LVOT VTI) of approximately 11 cm. The inferior vena cava was not plethoric. Initial management included norepinephrine and cautious fluid administration.

Over the subsequent hours, vasopressor requirements progressively increased in an attempt to normalize arterial pressure, resulting in a rise in mean arterial pressure by over 10 mmhg. Despite apparent improvement in macrocirculatory pressure targets, peripheral perfusion worsened. Extremities became progressively colder, CRT increased to approximately 6 s, and urine output declined. Arterial waveform analysis demonstrated progressive narrowing of pulse pressure, while repeat echocardiography showed a reduction in LVOT VTI, suggesting impaired forward flow despite higher arterial pressure.

This pattern illustrates Type 2 hemodynamic incoherence, in which impaired tissue perfusion results not from intrinsic microvascular dysfunction, but from hemodynamic constraints—resulting from imprecise therapy—limiting effective transmission of flow across the circulation. In this case, escalating vasopressor therapy likely increased vascular tone to a degree that promoted excessive arteriolar constriction and impaired capillary recruitment. As vascular tone approaches critical closing pressure (CCP), capillary segments become increasingly vulnerable to intermittent collapse or cessation of flow despite apparently adequate upstream pressure.

At the same time, increased afterload may further impair left ventricular forward flow, particularly in patients with limited cardiac reserve. Under these conditions, increases in arterial pressure may paradoxically reduce effective tissue perfusion because circulatory energy is no longer effectively transmitted through the capillary network.

Interface analysis: The dominant abnormalities in this phenotype involve both Interface I and Interface II. At Interface I, excessive afterload impairs effective left ventricular–arterial coupling and reduces forward flow despite higher arterial pressure. At Interface II, excessive vasoconstrictive tone increases resistance at the arteriolar–capillary transition and essentially causes uncoupling. Interface III is not the dominant mechanism in this example because there is no major evidence of venous congestion, while Interface IV may contribute secondarily if right ventricular afterload increases substantially during the course of illness. In this situation, norepinephrine would not necessarily be discontinued, particularly if required to maintain coronary and cerebral perfusion. Rather, the dose and arterial pressure target should be reassessed while determining whether forward flow can be improved through correction of preload limitation, treatment of myocardial dysfunction, reduction in avoidable afterload, or, in selected cases, inotropic or mechanical circulatory support.

Recognition of this phenotype is clinically important because further escalation of vasopressor therapy may worsen rather than improve tissue perfusion. Initial norepinephrine was appropriate to restore a critically low perfusion pressure. The concern arose not from its initiation, but from progressive escalation despite worsening peripheral perfusion, narrowing pulse pressure, and a fall in LVOT VTI. Restoration of coherence may instead require optimization of forward flow, reduction in excessive vasoconstrictive tone, reassessment of acceptable perfusion targets, mechanical circulatory support and careful integration of bedside perfusion indices rather than reliance on arterial pressure alone.

Take-home message: When increasing arterial pressure is accompanied by falling VTI, narrowing pulse pressure, and worsening peripheral perfusion, the vasopressor strategy should be reassessed rather than automatically escalated.

### 5.3. Type 2 Hemodynamic Incoherence: Increased Venous Congestion

A 66-year-old woman with severe pulmonary hypertension and right ventricular dysfunction was admitted with septic shock secondary to biliary sepsis. Initial resuscitation included fluid administration and norepinephrine, resulting in restoration of mean arterial pressure to approximately 70 mmHg. Despite normalization of arterial pressure, the patient developed progressive oliguria, rising lactate levels, worsening peripheral edema, and persistent prolongation of capillary refill time.

Point-of-care ultrasound demonstrated severe right ventricular dilation with reduced tricuspid annular plane systolic excursion (TAPSE), septal flattening, and a plethoric inferior vena cava with minimal respiratory variation. Venous Doppler examination revealed marked pulsatility of the portal vein and abnormal intrarenal venous flow patterns consistent with significant systemic venous congestion and elevated VExUS score. Lung ultrasound demonstrated progressive mild interstitial edema. Left ventricular systolic function was relatively preserved, but LVOT velocity time integral remained modest despite apparently adequate arterial pressure.

This pattern illustrates a second form of Type 2 hemodynamic incoherence, in which impaired tissue perfusion results from downstream hemodynamic constraints rather than primary microvascular dysfunction. In this setting, increased venous pressure and impaired venous drainage elevate capillary hydrostatic pressure, promoting transcapillary filtration and interstitial fluid accumulation. As interstitial pressure rises, diffusion distances between capillaries and tissues increase, while external compressive forces may impair both capillary and lymphatic patency and reduce effective microvascular flow transmission and lymphatic function.

Interface analysis: The dominant abnormalities in this phenotype involve Interface III and Interface IV. Interface IV dysfunction is reflected by impaired right ventricular–pulmonary arterial coupling with consequent elevation of systemic venous pressures. This produces secondary uncoupling at Interface III, where elevated venous and interstitial pressures impair effective capillary drainage and results in abnormal transcapillary flow. Interface I is relatively preserved in this example, while Interface II dysfunction is secondary rather than primary, resulting from mechanical and hemodynamic constraints imposed by congestion and elevated downstream pressures rather than intrinsic microvascular pathology.

Importantly, restoration of arterial pressure alone does not correct the underlying problem because the limitation resides in impaired transmission of flow across the capillary and venous compartments. Forward circulatory energy becomes progressively dissipated within the congested venous and interstitial system, resulting in apparent macro–microcirculatory uncoupling despite acceptable macrocirculatory variables.

Recognition of this phenotype has important therapeutic implications because additional fluid administration or excessive pressure augmentation may further worsen congestion and impair tissue perfusion. Restoration of coherence may instead require reduction in venous congestion, optimization of right ventricular function, improvement in forward flow, and careful management of fluid balance. In such cases, bedside assessment of venous physiology may provide more clinically relevant information than arterial pressure targets alone.

Take-home message: In right ventricular failure with systemic venous congestion, restoration of arterial pressure does not ensure tissue perfusion if impaired venous drainage remains the dominant constraint.

## 6. Clinical Relevance of Distinguishing Primary from Secondary Incoherence

The distinction between primary and secondary forms of hemodynamic incoherence is not merely semantic. At the bedside, the same clinical signals—persistent mottling, prolonged capillary refill time, oliguria, or rising lactate despite an acceptable arterial pressure—may lead to very different therapeutic decisions depending on the dominant mechanism. If the problem is interpreted as primary microcirculatory failure, the clinician may reasonably avoid further escalation of macrocirculatory targets and instead focus on source control, antimicrobial therapy, modulation of the inflammatory process, and time-dependent recovery of endothelial and microvascular function. Conversely, if the same findings are caused by secondary incoherence, then the abnormal tissue perfusion may remain correctable by addressing the hemodynamic constraint responsible for impaired flow transmission. This may include reducing excessive vasoconstrictive tone, improving forward flow, unloading venous congestion, optimizing right ventricular–pulmonary arterial coupling, or reconsidering fluid balance. In this sense, the classification proposed here is intended to function less as a diagnostic label and more as a bedside reasoning tool.

This distinction may also help explain why apparently similar resuscitation strategies can produce divergent responses between patients. A higher mean arterial pressure target may improve tissue perfusion in one patient if the limiting problem is inadequate perfusion pressure across an autoregulating vascular bed, yet worsen perfusion in another if the higher pressure is achieved primarily through excessive vasoconstriction, reduced stroke volume, or worsening venous congestion. Similarly, fluid administration may improve microcirculatory flow when preload limitation is the dominant constraint, but may worsen tissue oxygenation and organ function when the principal abnormality is venous congestion, interstitial edema, or right ventricular–pulmonary uncoupling. The relevant clinical question is therefore not whether pressure, flow, or volume is generally beneficial, but whether a specific intervention improves effective flow transmission across the dominant impaired interface in that patient at that moment.

The four-interface model provides a practical structure for this reasoning because it forces the clinician to ask where circulatory energy is being generated, transmitted, dissipated, or obstructed [3]. Interface I focuses on ventricular–arterial interaction and forward flow. Interface II focuses on the arteriolar–capillary transition and the distribution of flow into exchange vessels. Interface III emphasizes that tissue perfusion is not determined only by inflow, but also by venous drainage, capillary pressure, interstitial pressure, and lymphatic clearance. Interface IV highlights the dependence of systemic venous drainage and left-sided filling on right ventricular–pulmonary arterial coupling. By mapping incoherence to one or more of these interfaces, the clinician can move beyond the broad statement that the macrocirculation and microcirculation are dissociated and toward a more actionable explanation of why tissue perfusion remains abnormal.

This approach also supports a more dynamic view of resuscitation. The dominant mechanism of incoherence may change during the course of illness or as a consequence of therapy. A patient may initially present with primary septic microvascular dysfunction, later develop excessive vasoconstrictive constraint after escalating vasopressor therapy, and subsequently become congested after liberal fluid administration or evolving right ventricular dysfunction. Repeated bedside reassessment is therefore essential. Hemodynamic coherence should not be considered a fixed property of a patient, but rather a time-sensitive relationship between systemic hemodynamics, microvascular perfusion, and the interventions being applied.

Fluids and vasopressors remain lifesaving components of evidence-based shock resuscitation when used for appropriate indications. The proposed framework is not intended to replace established resuscitation protocols, but to help clinicians recognize when continued escalation of a generally appropriate therapy is no longer improving—and may be worsening—the physiological abnormality present in an individual patient.

The proposed assessment does not require a complete echocardiographic examination in every patient, but it does require competence in the focused measurements being used. Findings that are technically uncertain, internally inconsistent, or suggest complex structural or valvular disease should prompt comprehensive echocardiography or additional hemodynamic assessment.

## 7. Limitations

This framework has several limitations. It is a conceptual classification that has not yet undergone prospective validation, and its ability to improve clinical outcomes remains unknown. Identification of a dominant interface may be difficult in mixed shock states, in which several abnormalities coexist and change over time. Some components depend on focused ultrasound and therefore remain influenced by operator expertise, image quality, and interobserver variability.

## 8. Future Directions

Future research in shock physiology and resuscitation should increasingly incorporate mechanistic phenotyping of hemodynamic incoherence rather than treating macro–microcirculatory uncoupling as a physiologically uniform entity. Many contemporary studies evaluating resuscitation strategies continue to classify patients primarily according to global macrocirculatory variables or nonspecific markers of tissue perfusion, without distinguishing whether impaired microvascular function reflects primary microcirculatory pathology or secondary hemodynamic constraints across hemodynamic interfaces. As a result, fundamentally different physiologic states may be grouped together under the same conceptual definition of “incoherence,” potentially obscuring therapeutic signals and contributing to heterogeneous responses in interventional trials.

Future physiologic and interventional studies should therefore aim not only to determine whether coherence is present or absent, but also to characterize the dominant mechanism responsible for impaired tissue perfusion. Integration of bedside perfusion markers with assessment of the hemodynamic conditions that may produce secondary incoherence could allow more precise classification of persistent tissue hypoperfusion and improve interpretation of responses to resuscitation.

Future studies should evaluate whether distinguishing primary from secondary hemodynamic incoherence is feasible, reproducible, and clinically meaningful across different shock states. Prospective investigations should determine how frequently Type 1 and Type 2 phenotypes occur, how they overlap or evolve during resuscitation, and whether they are associated with different responses to fluids, vasopressors, inotropes, decongestive strategies, or changes in perfusion targets. Studies should also assess whether combining serial tissue-perfusion findings with conventional and advanced hemodynamic monitoring improves the identification of the mechanism underlying persistent incoherence. Multimodal analytical approaches, including artificial intelligence-assisted phenotyping, may eventually help integrate these dynamic data, but would require transparent physiological interpretation and external validation. Ultimately, interventional studies will be needed to determine whether treatment guided by the mechanism of incoherence improves clinically relevant outcomes.

## 9. Conclusions

Hemodynamic incoherence is not a physiologically uniform phenomenon. Apparent dissociation between macro- and microcirculatory variables may arise either from primary microvascular dysfunction or from impaired transmission of flow across upstream or downstream hemodynamic interfaces. Recognition of this distinction is clinically important because therapeutic approaches may differ substantially between these phenotypes.

The four-interface model provides a structured physiologic framework for understanding these mechanisms at the bedside. Integration of simple clinical examination with focused ultrasound may help identify the dominant interface at which circulatory energy transfer becomes impaired and may improve physiologic interpretation during resuscitation. Proper phenotyping of incoherence may help clinicians avoid unnecessary or potentially harmful escalation of hemodynamic interventions when restoration of coherence depends primarily on resolution of the underlying pathophysiology rather than further increases in pressure or flow.

## Figures and Tables

**Table 1:** Bedside assessment of the four hemodynamic interfaces and potential therapeutic implications.

Interface	Dominant Question	Bedside Clues	Possible Intervention
I	Is forward flow limited by LV/arterial coupling?	Low VTI, narrow pulse pressure, poor EF/VA coupling	inotrope, reduce afterload, MCS
II	Is arteriolar/capillary distribution failing?	CRT/mottling, microvascular heterogeneity	source control, avoid excessive vasopressor, test flow/pressure response
III	Is venous drainage/congestion limiting tissue flow?	VExUS, edema, oliguria, high CVP/JVP	decongestion, reduce fluid, RV optimization
IV	Is RV–pulmonary coupling limiting systemic drainage?	RV dilatation, septal flattening, low TAPSE/FAC/S′	reduce RV afterload, optimize ventilation, inotrope/vasopressor balance

## Data Availability

No new data were created or analyzed in this study.

## References

[1] Ince C. (2005). The microcirculation is the motor of sepsis. Crit. Care.

[2] Ince C. (2015). Hemodynamic coherence and the rationale for monitoring the microcirculation. Crit. Care.

[3] Rola P., Kattan E., Siuba M.T., Haycock K., Crager S., Spiegel R., Hockstein M., Bhardwaj V., Miller A., Kenny J.E. (2025). Point of View: A Holistic Four-Interface Conceptual Model for Personalizing Shock Resuscitation. J. Pers. Med..

[4] Miller A., Kirk-Bayley J., Peck M., Wilkinson J. (2026). Energy, flow and pressure in the cardiovascular system: A narrative review of how the circulation works. Anaesthesia.

[5] De Backer D., Donadello K., Taccone F.S., Ospina-Tascon G., Salgado D., Vincent J.L. (2011). Microcirculatory alterations: Potential mechanisms and implications for therapy. Ann. Intensive Care.

[6] Ospina-Tascon G., Neves A.P., Occhipinti G., Donadello K., Büchele G., Simion D., Chierego M.L., Silva T.O., Fonseca A., Vincent J.L. (2010). Effects of fluids on microvascular perfusion in patients with severe sepsis. Intensive Care Med..

[7] Yeh Y.C., Chiu C.T. (2019). Association and dissociation of microcirculation and macrocirculation in critically ill patients with shock. J. Emerg. Crit. Care Med..

[8] Jacquet-Lagrèze M., Boisseau M., Bouhamri N., Schweizer R., Bidar F. (2026). Passive leg raising, peripheral perfusion, and cardiac output: Insights into hemodynamic coherence. Crit. Care.

[9] Jacquet-Lagrèze M., Bouhamri N., Portran P., Schweizer R., Baudin F., Lilot M., Fornier W., Fellahi J.L. (2019). Capillary refill time variation induced by passive leg raising predicts capillary refill time response to volume expansion. Crit. Care.

[10] Hernández G., Valenzuela E.D., Kattan E., Castro R., Guzmán C., Kraemer A.E., Sarzosa N., Alegría L., Contreras R., Oviedo V. (2024). Capillary refill time response to a fluid challenge or a vasopressor test: An observational, proof-of-concept study. Ann. Intensive Care.

[11] The ANDROMEDA-SHOCK-2 Investigators for the ANDROMEDA Research Network, Spanish Society of Anesthesiology, Reanimation and Pain Therapy (SEDAR), and Latin American Intensive Care Network (LIVEN) (2025). Personalized Hemodynamic Resuscitation Targeting Capillary Refill Time in Early Septic Shock: The ANDROMEDA-SHOCK-2 Randomized Clinical Trial. JAMA.

[12] Miller A., Rola P., Spiegel R., Haycock K. (2026). Arteriolar Collapse and Haemodynamic Incoherence in Shock: Rethinking Critical Closing Pressure. J. Pers. Med..

[13] Beaubien-Souligny W., Rola P., Haycock K., Bouchard J., Lamarche Y., Spiegel R., Denault A.Y. (2020). Quantifying systemic congestion with point-of-care ultrasound: Development of the VExUS grading system. Ultrasound J..

